# C-Peptide Reduces Mitochondrial Superoxide Generation by Restoring Complex I Activity in High Glucose-Exposed Renal Microvascular Endothelial Cells

**DOI:** 10.5402/2012/162802

**Published:** 2012-06-21

**Authors:** Himani Vejandla, John M. Hollander, Anand Kothur, Robert W. Brock

**Affiliations:** ^1^Department of Physiology and Pharmacology, West Virginia University School of Medicine, P.O. Box 9105, Morgantown, WV 26506, USA; ^2^Center for Cardiovascular and Respiratory Sciences, West Virginia University School of Medicine, Morgantown, WV 26506, USA; ^3^Division of Exercise Physiology, West Virginia University School of Medicine, Morgantown, WV 26506, USA

## Abstract

Hyperglycemia-mediated microvascular damage has been proposed to originate from excessive generation of mitochondrial superoxide in endothelial cells and is the suggested mechanism by which the pathogenesis of diabetes-induced renal damage occurs. C-peptide has been shown to ameliorate diabetes-induced renal impairment. Yet, the mechanisms underlying this protective benefit remain unclear. The objective of this study was to determine whether C-peptide affords protection to renal microvascular endothelial cell mitochondria during hyperglycemia. Conditionally immortalized murine renal microvascular endothelial cells (MECs) were exposed to low (5.5 mM) or high glucose (25 mM) media with either C-peptide (6.6 nM) or its scrambled sequence control peptide for 24 or 48 hours. Respiratory control ratio, a measure of mitochondrial electrochemical coupling, was significantly higher in high glucose renal MECs treated with C-peptide than those of high glucose alone. C-peptide also restored high glucose-induced renal MEC mitochondrial membrane potential changes back to their basal low glucose state. Moreover, C-peptide prevented the excessive mitochondrial superoxide generation and concomitant reductions in mitochondrial complex I activity which are mediated by the exposure of the renal MECs to high glucose. Together, these data demonstrate that C-peptide protects against high glucose-induced generation of mitochondrial superoxide in renal MECs via restoration of basal mitochondrial function.

## 1. Introduction 

Diabetic renal impairment, a serious and frequent complication of both type I and type II diabetes [1, 2], is a leading cause of chronic renal disease [3, 4]. The pathobiology of diabetic renal impairment is founded in the microcirculation. It is well established that the chronic hyperglycemia associated with diabetes directly damages the microcirculation, leading to small vessel dysfunction [5]. More specifically, hyperglycemia has been shown to selectively damage endothelial cells because their glucose transport rate is not diminished, leading to an excessive accumulation of glucose within them [6, 7]. As a consequence, the elevated intracellular glucose that ensues leads to increased generation of mitochondrial superoxide, proposed as a central player in the pathogenesis of all diabetic complications [6]. Several reports contend that the diabetic state increases mitochondrial superoxide generation both in *in vitro* high glucose conditions [8–11] as well as in diabetic animal models [12, 13]. Furthermore, this excessive superoxide generation leads to the subsequent accumulation of other free radicals, thereby imparting diffuse damage to proteins and mitochondrial DNA, and ultimately leading to mitochondrial dysfunction [14, 15]. 

Complex I is the first member of the mitochondrial membrane-bound electron transport chain and its substrates/cofactors have the lowest potentials in the chain [16]. Inhibition of the electron transport chain during pathological conditions and/or the build-up of NADH (and subsequent elevation in the NADH/NAD^+^ ratio) has been shown to contribute significantly to superoxide generation by complex I [17]. Under physiological conditions, mitochondrial superoxide originates predominantly from complex I and complex III [18, 19], yet growing evidence suggests that complex I alone is responsible for the majority of the superoxide produced within intact mammalian mitochondria [20]. 

C-peptide, a byproduct of insulin biosynthesis [21], had long been thought to be physiologically inert. However, several studies have reported the beneficial effects of exogenous C-peptide on renal function in type 1 diabetes [22, 23]. In fact, short-term infusions of C-peptide have been shown to improve glomerular hyperfiltration and reduce albuminuria [24–26], whereas long-term administration improved overall renal function in diabetic patients [27, 28]. Not surprisingly, many *in vitro *studies have been aimed at delineating the molecular and cellular basis for the observed actions of C-peptide [29–31]. Despite these reports, the exact nature of its bioactivity and physiologic impact on the renal microcirculation during diabetes needs further clarification. Given that C-peptide, administered in the absence of insulin, provides benefits to general renal and microvascular function during type I diabetes, our central hypothesis follows that C-peptide independently affords protection to high-glucose exposed renal microvascular endothelial cells (MECs) by diminishing mitochondrial superoxide generation via restoration of basal mitochondrial complex I activity. To examine this, we evaluated the effects of C-peptide treatment on renal MEC mitochondrial respiration, membrane potential changes, superoxide production, and complex activity while exposed to a high glucose environment.

## 2. Materials and Methods 

Biosynthetic C-peptide (NH_2_-EVEDPQVAQLELGGGPGAGDLQTLALQVARQ-COOH) and its scrambled sequence peptide control (NH_2_-LGGGPQVGTLLAQVEQEAEDGLDVRLAQQPA-COOH), both with a purity greater than 90% (HPLC), was synthesized by PrimmBiotech, Inc. (Cambridge, MA). The scrambled sequence peptide control has the same composition as the native C-peptide, but with the amino acid residues in random order. Cell culture media was supplied by Gibco (Invitrogen, Carlsbad, CA). All other chemicals were from Sigma Chemical (St. Louis, MO), with the exception of MitoSOX Red [3,8-phenanthradinediamine,  5-(6′  triphenylphosphoniumhexyl)-5,6  dihydro-6-phenyl], which was purchased from Molecular Probes (Invitrogen, Carlsbad, CA).

### 2.1. Cell Culture

Conditionally immortalized renal microvascular endothelial cells (MECs) were obtained from Dr. Isaiah J. Fidler (MD Anderson Cancer Center). Cells were derived from the kidneys of transgenic mice harboring the temperature-sensitive SV-40 large T antigen, referred to as *H-2Kb-tsA58 *mice [32]. Cells were either grown in a T75 flask or 100 mm plates in a humidified incubator gassed with 5% CO_2_ and 95% O_2_ in DMEM containing 5% fetal bovine serum and supplemented with nonessential amino acids, penicillin, and streptomycin. Cells were used between passages 3–10 and renal MECs were grown and maintained at 33°C. Once the cells reached 50–60% confluence, they were shifted to 37°C to permit activation of the temperature sensitive antigen and retention of the original endothelial cell phenotype. After 48 hours at 37°C, cells were serum deprived for 24 hours to allow for synchronization of cell cycles prior to treatments. Cells were subsequently divided into the following groups and medium was supplemented with the following agents: (A) low-glucose—5.5 mM D-glucose (LG); (B) low-glucose with C-peptide—6.6 nM (LG + C-pep); (C) high-glucose—25 mM D-glucose (HG); (D) high-glucose with C-peptide—6.6 nM (HG + C-pep); (E) low-glucose with scrambled peptide, 6.6 nM and (F) high glucose with scrambled peptide for either 24- or 48-hours. Low glucose cells were also treated with 19.5 mM raffinose to serve as an osmotic control. We chose to treat cells with 6.6 nM of C-peptide because that is the physiological postprandial concentration observed in humans [33] and this concentration is comparable to the previous literature involving *in vitro* C-peptide studies [34]. For the sake of clarity and brevity, we did not present data on the high-glucose/low-glucose + scrambled peptide control groups since they did not differ from their respective nontreatment conditions for any of the parameters assessed. Renal MECs treated with high-glucose did not exhibit any changes in cell viability (as assessed by trypan blue exclusion), nor did they demonstrate changes in their characteristic cobblestone morphology (data not shown). 

### 2.2. Mitochondrial Respiration Assays

Mitochondrial respiration (a marker for mitochondrial electrochemical coupling) was measured by oxygen consumption rates in digitonin-permeabilized renal MECs using a Clark-type oxygen electrode [14]. Cells were trypsinized, centrifuged at 1000 g for 10 min and resuspended in 10 mL of respiration buffer containing (in mmol/L) 20 HEPES, 10 MgCl_2_, and 250 sucrose with 100–150 *μ*g of digitonin. Cells were then incubated on ice for 10 minutes, washed three times with respiration buffer containing 0.5% bovine serum albumin, followed by resuspension at 5–8 million cells per 1.5 mL of respiration buffer into a Gilson chamber (Gilson; Middleton, WI). The chamber was maintained at 30°C and attached to YSI 5300 Biological Oxygen Monitor (YSI; Yellow Springs, OH) [35]. After an initial period of stabilization, the substrates glutamate and malate (100 mM of each) were added followed by ADP. Protein content was determined using the Bradford method (BCA Protein assay, Pierce), and values were expressed as nmol O_2_ consumed/min/mg protein. State 3 respiration is an active state of maximal respiration with ADP and substrates, while state 4 respiration is a slow respiration state with low ADP and substrates [36]. The respiratory control ratio (RCR), a ratio of the state 3 to state 4 respiration rates, is a measure coupling the transfer of electrons driving oxygen consumption and proton transfer driving ATP synthesis.

### 2.3. Mitochondrial Membrane Potential Measurements

Assessments of mitochondrial membrane potential (ΔΨm) were performed using a previously described method [37], which employs the J-aggregate lipophilic cationic dye, JC-1 (Molecular Probes; Eugene, OR). Briefly, renal MECs were incubated in the dark with JC-1 (7.5 *μ*M, 30 min at 37°C) following either the 24- or 48-hour treatments. After incubation, renal MECs were measured for fluorescence emission at 530 nm (monomer = green) and at 590 nm (J-aggregates = red) in a F-2500 Fluorescence Spectrophotometer (Hitachi High Technologies America; Pleasanton, CA) equipped with a cover slip holder and using an excitation wavelength of 488 nm. Relative ΔΨm changes were calculated using the ratio of the J-aggregate to monomer (590 nm/530 nm). Values were expressed as fold increase in J-aggregate/monomer fluorescence over control cells. As a complement, cells were grown on six-well dishes and observed using an Eclipse 800 Fluorescence Microscope (Nikon; Melville, NY) equipped with a dual filter for fluorescein and rhodamine. 

### 2.4. Mitochondrial Superoxide Measurements

Mitochondrial superoxide was measured using a previously described flow cytometry method [38]. Briefly, after the 24- or 48-hour treatments, MitoSOX Red was added to fresh media to a final concentration of 5 **μ**M according to manufacturer's instructions. The renal MECs were incubated with the MitoSOX for 30-minutes in the dark, washed two times with PBS containing calcium and magnesium. Antimycin (100 *μ*M), a complex III inhibitor, was used as a control. Flow cytometry measurements were carried out using a FACScalibur (BD Biosciences; San Jose, CA). A laser at 488 nm excited MitoSOX Red, and data was collected at forward and side scatter, 582/42 nm (FL2) channels. Data were presented in the FL2 channel. Cell debris, represented by a distinct low forward scatter, was gated out of the analyses. 

### 2.5. Mitochondrial Complex Activity Measurements

Mitochondrial complex I, III, and IV activities were assessed using the previously described method of Trounce et al. [39]. Complex activities were expressed as nmol/min/mg protein.

### 2.6. Western Blotting

Cell supernatants were obtained from the renal MECs grown in 100 mm plates and subjected to 24-hour treatments. While adherent, cells were washed twice with ice-cold PBS followed by treatment with ice-cold 1X RIPA Buffer (20 mM Tris-HCl (pH 7.5), 150 mM NaCl, 1 mM Na_2_EDTA, 1 mM EGTA, 1% NP-40, 1% sodium deoxycholate, 2.5 mM sodium pyrophosphate, 1 mM beta-glycerophosphate, 1 mM Na_3_VO_4_, 1 *μ*g/mL leupeptin) supplemented with protease and phosphatase inhibitor cocktails, for 5-minutes on ice. Renal MECs were scraped gently using a rubber policeman, collected into a 1.5 mL centrifuge tube and sonicated briefly to ensure complete lysis of cells. The extract is then centrifuged for 10 minutes at 14,000 g to obtain the supernatants. Protein concentration was determined using Pierce BCA protein assay kit (Thermo Fisher Scientific Inc., Rockford, IL)

Cell supernatants (equivalent to 40 mg of protein) were separated on a 4–12% Novex NuPAGE Bis-Tris gels using Novex system (Invitrogen, Carlsbad, CA) and then transferred on to nitrocellulose membrane. Membranes were then blotted for Extracellular-Signal Regulated Kinase (ERK)1/2 (p44/p42 [ERK1/2] 137F5 Rabbit mAb), Phospho ERK1/2 (p44/p42 [ERK1/2] (Thr202/Tyr204) Rabbit mAb), p38 Mitogen-Activated Protein Kinase (MAPK) mAb, and phospho p38 MAPK (Thr180/Tyr182) Rabbit mAb (Cell signaling, Danvers, MA), followed by incubation with peroxidase conjugated anti-rabbit secondary antibody. Membranes were developed with ECL advance detection reagent (Amersham, GE Healthcare, Piscataway, NJ) followed by detection of chemiluminescent signal using G:Box, Gel Imager (Syngene, Synoptics Ltd.). Densitometric analysis was performed using Gene Tools (Syngene) software and data were calculated as arbitrary units. Phosphorylated MAPK phosphorylations were normalized to total MAPK levels.

### 2.7. Statistical Analysis

Statistical significances were determined using standard ANOVA procedures. If significance was found, the effects were tested further by using the Student Newman-Keuls post hoc comparison. A probability of 0.05 was accepted as statistically significant, and the sample sizes for each experimental group provided a statistical power of greater than 80%. Data are expressed as means ± SEM.

## 3. Results

### 3.1. C-Peptide Ameliorated Defects in Mitochondrial Electrochemical Coupling during High Glucose Exposure of Renal MECs

**Figure 1:**
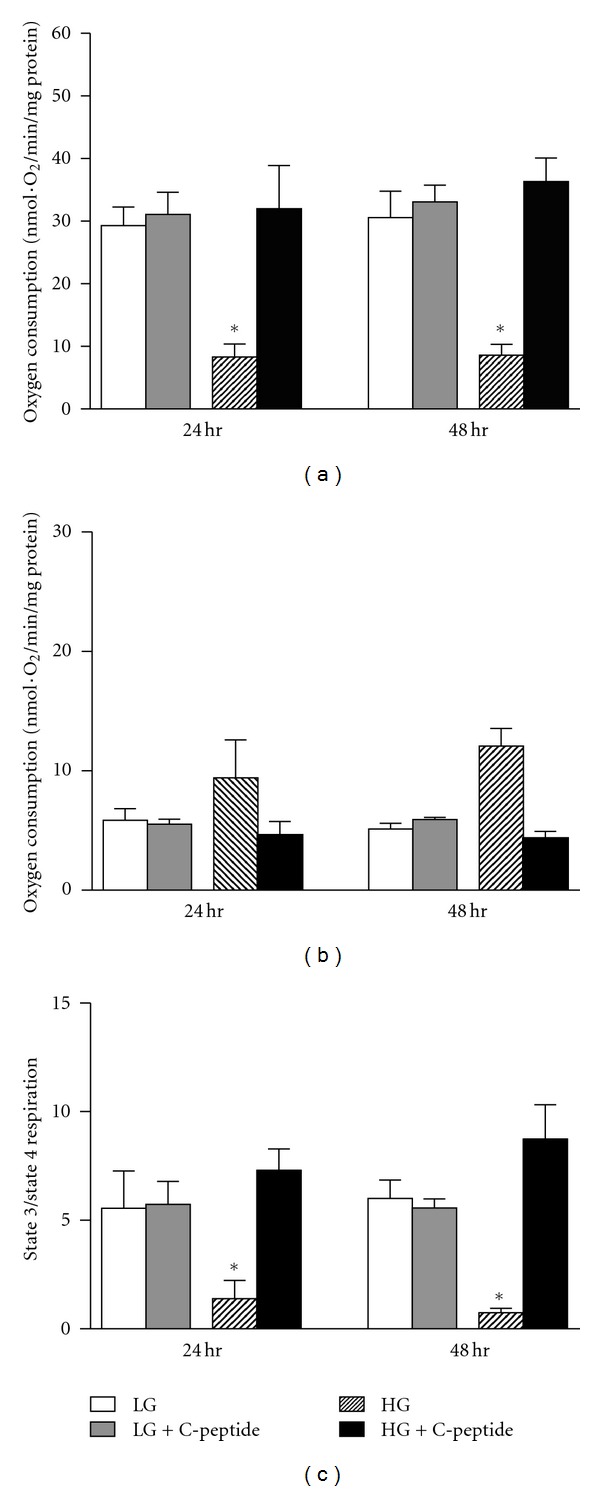
(a) State 3 respiration is the slope of measured O_2_ consumption in the presence of glutamate-malate and ADP. ∗, significantly different than low-glucose and high-glucose + C-peptide (*P* < 0.02). (b) State 4 respiration is the slope of measured O_2_ consumption after ADP depletion. (c) RCR is the ratio of state 3 to state 4 respiration. ∗, significantly less than low-glucose and high-glucose + C-peptide (*P < 0.0001). *n* = 8 experiments. Data represent mean ± SEM.

State 3 mitochondrial respiration is the slope of measured oxygen consumption in the presence of glutamate-malate and ADP. State 3 respiration rates were significantly decreased in high glucose cells at both 24- and 48-hours. C-peptide treatment restored state 3 respiration to basal levels (Figure 1(a); *P* < 0.02). State 4 respiration is the slope of measured oxygen consumption after ADP depletion. State 4 respiration was increased in high glucose cells at both 24- and 48-hours while C-peptide treatment restored state 4 respiration (Figure 1(b)). RCR, the ratio of state 3-to-state 4 respiration, is a measure coupling the transfer of electrons driving oxygen consumption and proton transfer driving ATP synthesis. RCRs were significantly decreased in high glucose cells at both 24- and 48-hours and were restored with C-peptide (Figure 1(c); *P* < 0.0001). These data demonstrate that C-peptide abrogates mitochondrial respiratory defects caused by high glucose.

### 3.2. C-Peptide Restored the Mitochondrial Membrane Potential (ΔΨm) of Renal MECs during High Glucose Exposure

**Figure 2:**
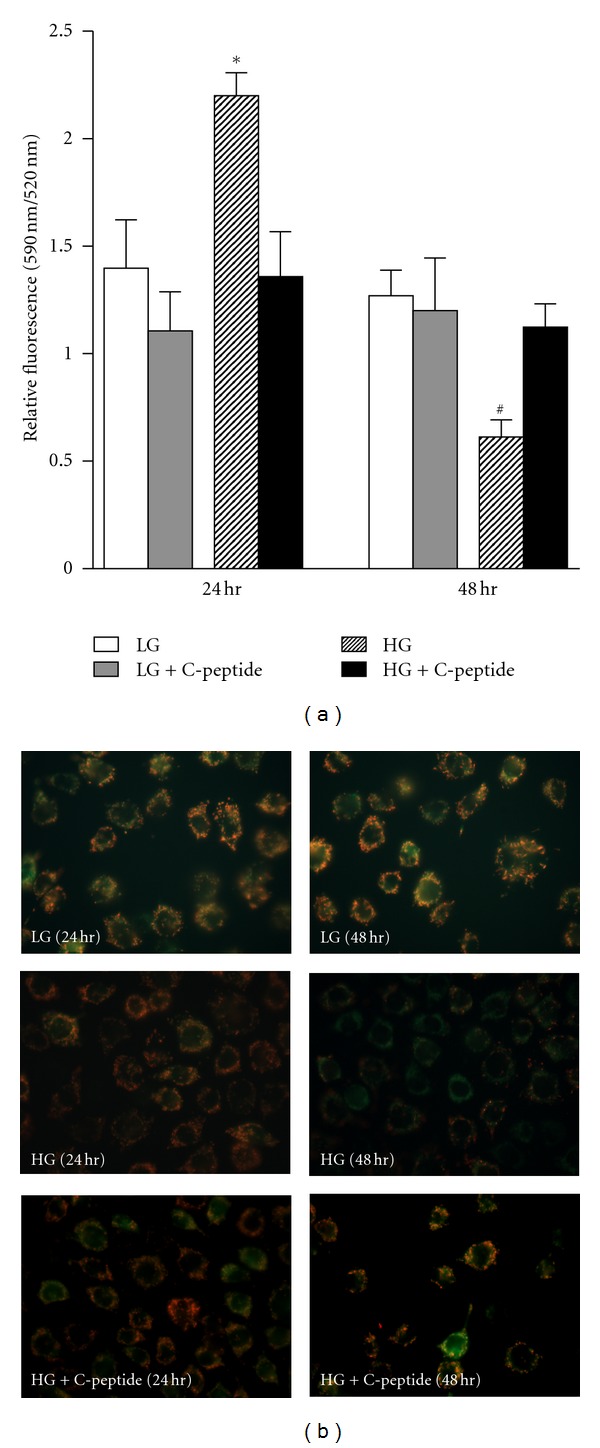
(a) At 24 hours, high glucose resulted in significant mitochondrial membrane hyperpolarization. Subsequently, endothelial mitochondria were depolarized (after 48 hours). Treatment with C-peptide resulted in restoration of ΔΨm at both 24 and 48 hours of exposure to a high glucose stress. ∗, significantly greater than low-glucose and high-glucose + C-peptide (*P* < 0.009); #, significantly less than low-glucose and high-glucose + C-peptide (*P* < 0.002). Data represent means ± SEM and *n* = 6 experiments. (b) Representative fluorescence micrographs of renal MECs treated with JC-1.

Changes in ΔΨm were measured in renal MECs using JC-1 staining. At 24-hours, high glucose exposure resulted in significant hyperpolarization of the mitochondrial membrane (Figure 2; *P* < 0.009). Subsequently, endothelial mitochondria were depolarized after 48-hour (Figure 2; *P* < 0.002). Treatment with C-peptide resulted in restoration of ΔΨm at both 24- and 48-hours of exposure to a high glucose stress.

### 3.3. C-Peptide Reduced the Excessive Mitochondrial Superoxide Levels Observed in High Glucose Exposed Renal MECs

**Figure 3:**
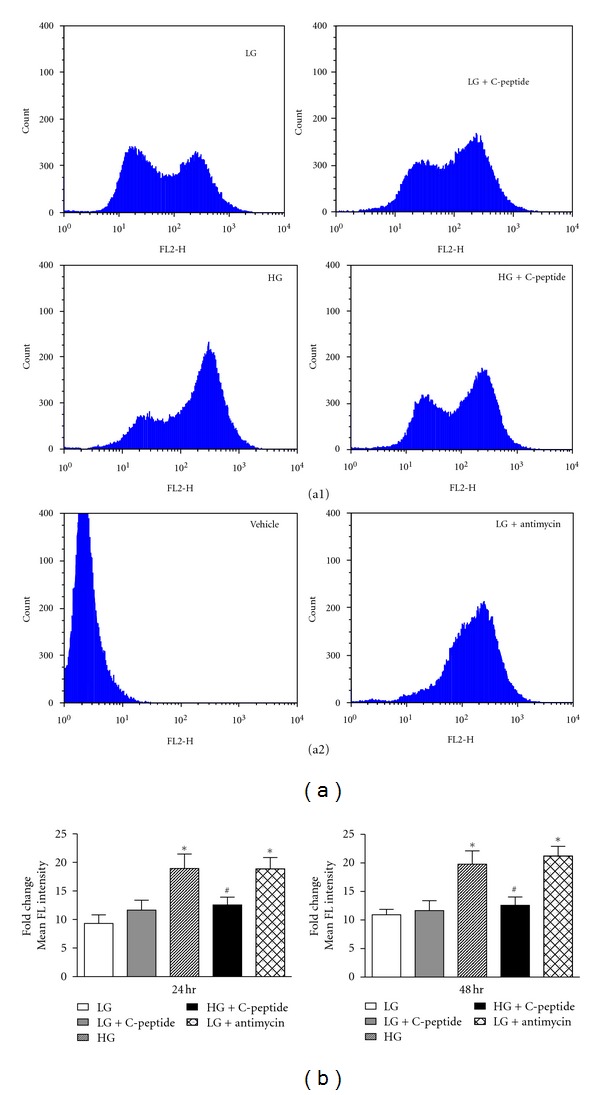
(a) Representative histograms of MitoSOX Red flow cytometry data at 24 hrs. *Y*-axes represent the number of counts of cells that emit fluorescence. *X*-axes represent the average fluorescence intensity of cells. (a1) represents treatment conditions; (a2) represent vehicle control and positive control. (b) Fold-change of mean fluorescence intensity of cells, including the control of low-glucose + antimycin. ∗, significantly different than low-glucose and high-glucose + C-peptide (*P* < 0.0001). #, significantly different than high-glucose and low-glucose + antimycin (*P* < 0.0001).

Using MitoSOX Red and measuring fluorescence by flow cytometry, the exposure of renal MECs to high glucose significantly elevated mitochondrial superoxide generation (Figure 3(b); *P* < 0.0001) compared to low glucose at both 24- and 48-hours. C-peptide treatment restored mitochondrial superoxide generation to basal levels at both time points.

### 3.4. C-Peptide Improves Mitochondrial Complex I Activity in High Glucose Exposed Renal MECs

**Figure 4:**
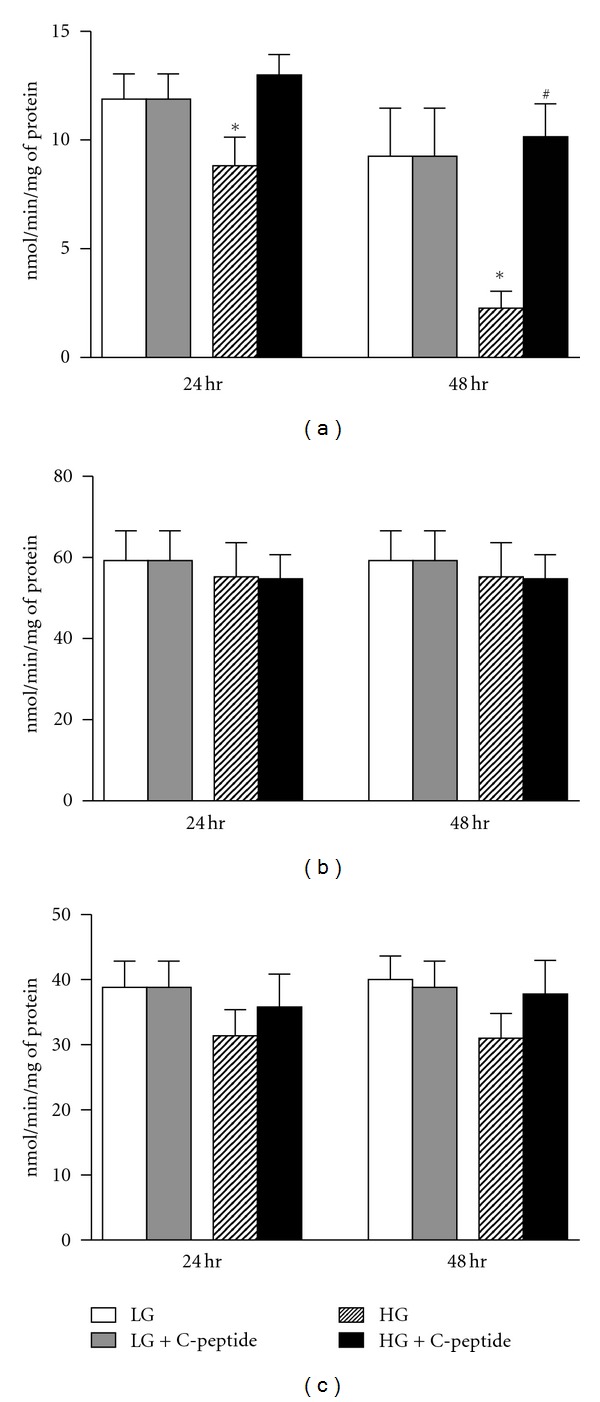
Mitochondrial complex activity, as measured by spectrophotometry. (a) There is a significant decrease in complex I activity in high-glucose exposed renal MECs compared to low-glucose and high-glucose + C-peptide at both 24- and 48-hours. ∗, significantly less than low-glucose and high-glucose + C-peptide (*P* < 0.002 at 24 hr and *P* < 0.0009 at 48 hr). #, significantly greater than high glucose at 48 hr (*P* < 0.05). (b) and (c) Both complex III and IV activities are not different among the treatment conditions. Data represent mean ± SEM; *n* = 16 experiments.

Renal MECs exposed to high glucose exhibited significant reductions in complex I activity at 24- and 48-hours (Figure 4(a); (*P* < 0.002 at 24 hr and *P* < 0.0009 at 48 hr), with no change to complex III and IV (Figures 4(b) and 4(c)). C-peptide restored complex I activity back to levels observed with low glucose.

### 3.5. C-Peptide Activates Extracellular Signal Regulated Kinase (ERK) 1/2 in High Glucose Exposed Renal MECs

**Figure 5:**
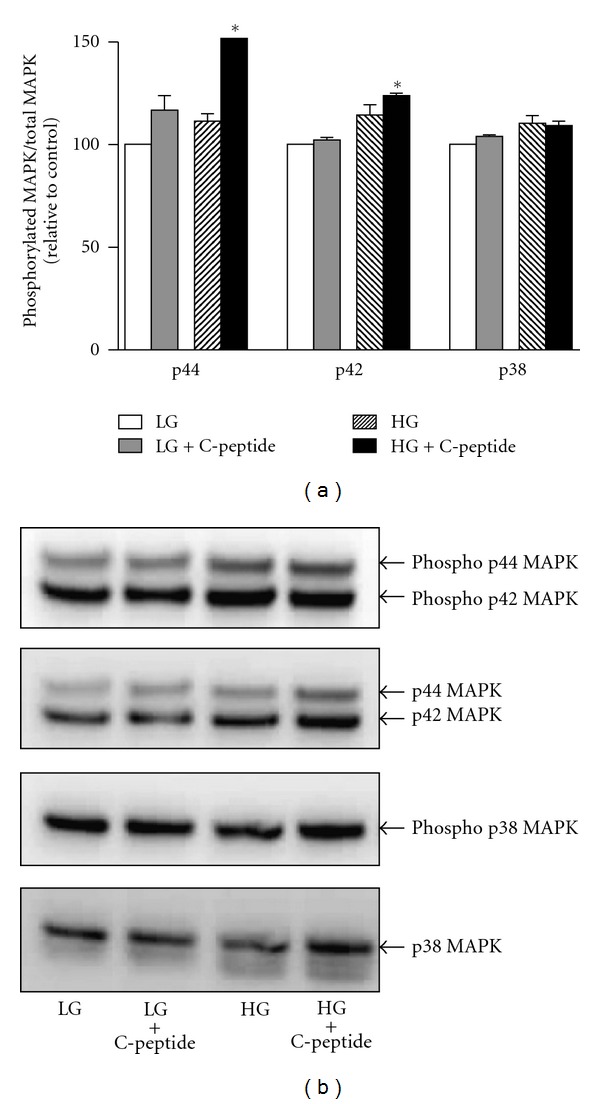
Cell supernatants obtained after 24 hr treatments were subjected to western blotting analysis for detection of phosphorylated ERK isoforms (p44 kDa/pERK1 and p42 kDa/pERK2) and Phosphorylated p38 MAPK isoforms. (a) Phosphorylated MAPK band intensities were normalized to total MAPK levels. *significantly greater than all other groups (*P* < 0.0005). Data are expressed as percent change relative to control low glucose treatment. Data represent mean ± SEM, *n* = 6. (b) Representative western blot images.

C-peptide treatment resulted in activation of ERK 1/2 (Figure 5(a); *P* < 0.0005) in high glucose exposed renal MECs, however it failed to induce phosphorylation of p38 MAPK.

## 4. Discussion

Chronic hyperglycemia is the main trigger initiating diabetic renal impairment. However, to varying degrees, chronic hyperglycemic injury affects all of the cell types within the glomerular, vascular and tubulointerstitial compartments of the kidney. However, the initial stages of diabetic renal impairment can be ascribed to dysfunction of glomerular capillaries and barrier function, which clinically manifest as hyperfiltration and microalbuminuria. The glomerulus is composed of endothelial cells, podocytes, and mesangial cells, all of which play a crucial role in maintaining barrier function. Furthermore endothelial cells, both from glomeruli and the tubulointerstitium, play an important role in the overt hyperglycemic damage to the kidney due to the previously discussed deficiency in glucose transport. The murine renal microvascular endothelial cells utilized in this study are an ideal cell type to study the effects of high glucose-induced deleterious changes that ultimately lead to renal microvascular endothelial dysfunction.

This study demonstrates, for the first time, that C-peptide confers protection against hyperglycemia-mediated mitochondrial dysfunction in endothelial cells. We show that C-peptide inhibits high glucose-induced changes to mitochondrial respiration, membrane potential, superoxide generation, and complex I activity. Several studies suggest that C-peptide prevents diabetes-induced renal microvascular complications, and subsequently improves renal function [24–28]. Although mitochondrial superoxide generation has been widely implicated in the pathogenesis of diabetic microvascular complications [5, 7], there is a dearth of knowledge regarding the impact of C-peptide on diabetes-induced mitochondrial dysfunction. This is the first investigation addressing whether the protection rendered by C-peptide during diabetes involves the mitochondria. 

Our studies examining the effect of C-peptide on hyperglycemia-mediated mitochondrial dysfunction suggest that C-peptide improved mitochondrial respiration in high glucose-exposed renal MECs. Mitochondrial respiration is a reflection of its capacity to synthesize ATP, which occurs by means of the electron transport chain and oxidative phosphorylation. The processes of electron transfer and ATP generation are tightly coupled, with respiratory control ratio used as an indicator of the extent of electrochemical coupling. Recent reports using the mitochondria isolated from diabetic animals demonstrated compromised mitochondrial respiration [12, 13, 40]. Recently, Palmeira et al. [14] reported impairment of mitochondrial respiration rates under similar high-glucose conditions in HepG2 cells. Clearly, there is a need to delineate the mechanisms underlying high glucose-mediated damage to microvascular endothelial cells, the main cell type associated with diabetic end-organ complications. Since it has already been shown that C-peptide prevents diabetes-induced renal microvascular complications [25, 26], restoration of mitochondrial respiration and attenuation of mitochondrial superoxide generation in high glucose-exposed renal MECs is a legitimate channel by which C-peptide can exert these beneficial renal effects during diabetes. 

It is well recognized that the major pathways involved in the pathogenesis of diabetic microvascular dysfunction are activated by hyperglycemia-mediated superoxide generation by the mitochondria [6]. Hyperglycemia results in the increased availability of reducing equivalents NADH and FADH_2_, which in turn lead to increased electron transfer through the mitochondrial electron transport chain. The overall rate of this electron transport is governed by mitochondrial respiratory control, which ultimately is the amplitude of the electrochemical trans-membrane proton gradient. In our studies, we believe that the high glucose environment generates a high transmembrane proton gradient and high ΔΨm, thereby prolonging the presence of the superoxide generating intermediate-ubiquinone [41, 42]. This is in agreement with the findings of others demonstrating changes in ΔΨm in high glucose-exposed cells [37, 43]. We also showed that C-peptide treatment prevented both the hyperpolarization, and the subsequent depolarization, in high glucose-exposed renal MECs. We propose that the increases in ΔΨm, described as hyperpolarization, may result in the partial inhibition of electron transport chain complexes leading to a large stimulation of mitochondrial superoxide generation. Our studies also show increased superoxide generation by the mitochondria in high glucose-exposed renal MECs, which was abrogated by treatment with C-peptide. A similar increase in mitochondrial superoxide generation upon exposure to high glucose has also been reported in various other cell types [8, 10, 11, 37, 43]. 

Although all of the complexes of the mitochondrial electron transport chain contribute to total mitochondrial superoxide generation, growing evidence now suggests that complex I produces most of the superoxide generated within intact mammalian mitochondria [44]. It is well known that complex II also has the capacity to produce superoxide, but this only occurs while in the presence of specific inhibitors and is not a physiologically relevant site of generation [45]. Complex III, on the other hand, has been shown to produce large amounts of superoxide, at least in the presence of antimycin [45]. To characterize the role of each individual complex in the generation of excessive mitochondrial superoxide in our high glucose model, we assessed the activities of complex I, III, and IV. As expected in our high glucose exposed renal MECs, we found a significant reduction in complex I activity, with no apparent changes to the activities of complex III and IV. This is in accordance with the observations of others [35, 43–45]. Further, Lambert and Brand [17] have shown that high superoxide generation by the mitochondria requires inhibition of complex I activity. Surprisingly, C-peptide prevented the reduction in complex I activity caused by the high glucose exposure, in the absence of any effect on basal complex III and IV activities. Moreover, increased mitochondrial superoxide generation exacerbates mitochondrial oxidative stress further and leads to conversion of the mitochondrial glutathione pool to glutathione disulfide [46]. Glutathionylation of complex I has been shown to contribute to oxidative damage to its subunits and loss of activity [47, 48]. 

C-peptide has been shown to be a potent stimulant of MAPKs [49–51]. Activation of ERK by C-peptide has been shown in numerous cell types [31, 52–54]. Our experiments to study the effect of C-peptide on MAPKs yielded similar results demonstrating the activation of ERK1/2. This is especially important because several recent studies indicate that ERK 1/2 may affect the mitochondrial activities by regulating the expression of mitochondrial proteins in the nucleus in addition to having intrinsic mitochondrial activities [55]. Specifically, ERK1/2 has been shown to localize to mitochondria in human alveolar macrophages and control mitochondrial membrane potential as well as ATP production [56]. However, the exact mechanisms by which ERK1/2 are translocated and regulated in the mitochondria are still obscure and are beyond the scope of our study.

In this work, we have shown that C-peptide protects renal MECs exposed to a high glucose environment from mitochondrial dysfunction via improvements to mitochondrial electrochemical coupling and reductions in mitochondrial superoxide generation through restoration of mitochondrial respiratory complex I activity. Together, these data not only support the continued thought that C-peptide treatment affords protection against high glucose environments, but that its unique impact on the mitochondria should be a mechanism pursued in more detail.

## 5. Conclusions

Our work demonstrates that C-peptide curtailed high glucose-induced mitochondrial functional impairments in renal MECs. Although interest in the physiologic benefits of C-peptide has persisted for more than two decades, C-peptide has yet to make its way into standard treatment regimens for various diabetic complications. The findings from our work have to be confirmed *in vivo*, but provides proof-of-principle evidence in support of the inclusion of C-peptide to the existing therapeutic regimen for treatment of diabetic complications, specifically those related to diabetes-induced renal impairment.
